# Amphiphilic polyanhydride-based recombinant MUC4β-nanovaccine activates dendritic cells

**DOI:** 10.18632/genesandcancer.189

**Published:** 2019-05

**Authors:** Kasturi Banerjee, Shailendra K. Gautam, Prakash Kshirsagar, Kathleen A. Ross, Gaelle Spagnol, Paul Sorgen, Michael J. Wannemuehler, Balaji Narasimhan, Joyce C. Solheim, Sushil Kumar, Surinder K. Batra, Maneesh Jain

**Affiliations:** ^1^ Department of Biochemistry and Molecular Biology, University of Nebraska Medical Center, Omaha, NE, USA; ^2^ The Fred and Pamela Buffett Cancer Center, University of Nebraska Medical Center, Omaha, NE, USA; ^3^ Department of Chemical and Biological Engineering, Iowa State University, Ames, IA, USA; ^4^ Department of Veterinary Microbiology and Preventive Medicine, Iowa State University, Ames, IA, USA; ^5^ Nanovaccine Institute, Ames, IA and Omaha, NE, USA; ^6^ Eppley Institute for Research in Cancer and Allied Diseases, University of Nebraska Medical Center, Omaha, NE, USA

**Keywords:** MUC4, pancreatic cancer, cancer vaccine, immunotherapy, nanoparticle

## Abstract

Mucin 4 (MUC4) is a high molecular weight glycoprotein that is differentially overexpressed in pancreatic cancer (PC), functionally contributes to disease progression, and correlates with poor survival. Further, due to its aberrant glycosylation and extensive splicing, MUC4 is a potential target for cancer immunotherapy. Our previous studies have demonstrated the utility of amphiphilic polyanhydride nanoparticles as a useful platform for the development of protein-based prophylactic and therapeutic vaccines. In the present study, we encapsulated purified recombinant human MUC4-beta (MUC4β) protein in polyanhydride (20:80 CPTEG:CPH) nanoparticles (MUC4β-nanovaccine) and evaluated its ability to activate dendritic cells and induce adaptive immunity. Immature dendritic cells when pulsed with MUC4β-nanovaccine exhibited significant increase in the surface expressions of MHC I and MHC II and costimulatory molecules (CD80 and CD86), as well as, secretion of pro-inflammatory cytokines (IFN-γ, IL-6, and IL-12) as compared to cells exposed to MUC4β alone or MUC4β mixed with blank nanoparticles (MUC4β+NP). Following immunization, as compared to the other formulations, MUC4β-nanovaccine elicited higher IgG2b to IgG1 ratio of anti-MUC4β-antibodies suggesting a predominantly Th1-like class switching. Thus, our findings demonstrate MUC4β-nanovaccine as a novel platform for PC immunotherapy.

## INTRODUCTION

Pancreatic cancer (PC) has a dismal prognosis with an overall survival rate of 8%, due to the limited efficacy of existing treatment modalities including surgery, chemotherapy, and radiation [1, 2]. Furthermore, PC has an elaborate immunosuppressive microenvironment comprised of high desmoplasia, immune-suppressive cells and an anti-inflammatory cytokine *milieu* [3]. Due to the high level of chemotherapy-induced toxicity, PC patients seldom benefit from chemotherapy. Recent studies have shown that immunotherapy-based strategies like cancer vaccines can provide therapeutic benefit by breaking the immunological tolerance to self-derived tumor associated antigens (TAAs) and overcoming immunosuppression, thereby improving the overall survival and quality of life [4, 5]. However, the development of efficacious anti-cancer vaccines is arduous due to the challenges in finding TAAs, as the majority of these antigens behave as “self”, and therefore, are immunologically ignored by the host immune system [4].

Mucins are high molecular-weight glycoproteins that are overexpressed on various epithelial surfaces for protection and lubrication. Several mucins are aberrantly overexpressed in pancreatic cancer where they play tumor-promoting role. Due to their aberrant expression and glycosylation, functional involvement in the pathogenesis and correlation with poor prognosis, mucin family members have emerged as ideal TAAs for PC and are currently being exploited for cancer immunotherapy [6]. Mucin1 (MUC1) is one of the well-studied targets for cancer vaccine development [7]. MUC1 peptide and glycopeptide-based vaccine studies have shown their potential in eliciting anti-tumor responses in various malignancies [8–13]. However, the limited immunogenic epitopes provided by peptide-based MUC1 vaccines have achieved suboptimal clinical success in PC patients [10, 14, 15]. Unlike MUC1, Mucin4 (MUC4) is undetectable in normal pancreatic tissue and its expression progressively increases with PC progression [16]. MUC4 is putatively cleaved at a Gly-Asp-Pro-His (GDPH) site in an autocatalytic manner, generating two subunits: a large N-terminal subunit called MUC4α that contains the characteristic tandem repeat domain, and a smaller membrane-tethered subunit termed MUC4β [17–19]. The MUC4β region is considered functionally important as it has 3 EGF-like domains that interact with HER-2 and promote cancer cell proliferation [6, 19, 32]. In a previous study, it was shown that the mice immunized with MUC4 glycopeptides conjugated to tetanus toxoid induced strong immune responses and predominantly produced IgG1 antibodies [20]. However, such “cherry-picked” immuno-dominant peptides limit the epitopes that can be employed to elicit immune responses in an unbiased manner, and thus are of limited translational value. While the large size of MUC4 can potentially provide a large epitope repertoire for eliciting potent immune responses, the production and purification of intact megadalton MUC4 protein is challenging. To circumvent these problems, this study investigated the utility of recombinant MUC4β subunit for tumor vaccine development.

One of the major challenges of vaccine delivery vehicles is to ensure protein stability and release over a sustained period [21, 22]. Amphiphilic polyanhydride nanoparticles (NPs), composed of 1,8-bis(*p*-carboxyphenoxy)-3,6-dioxaoctane (CPTEG) and 1,6-bis(*p*-carboxyphenoxy) hexane (CPH) have been shown to stabilize the structure and activity of encapsulated proteins while providing sustained release *via* a surface erosion mechanism [23, 24]. Furthermore, these NPs have been shown to be readily internalized by antigen presenting cells (APCs), such as dendritic cells (DCs) and macrophages, leading to the upregulation of cell surface activation markers including major histocompatibility complexes class I and II (MHC I and MHC II), co-stimulatory molecules (CD80, CD86, CD40), secretion of inflammatory cytokines and generation of humoral responses [25–28].

In the present study, we encapsulated endotoxin-free recombinant human MUC4β in 20:80 CPTEG:CPH NPs (MUC4β-nanovaccine). The relationships between antigen release kinetics, the ability of MUC4β-nanovaccine to activate APCs, and the nature of immune responses elicited were investigated. These studies demonstrated that the MUC4β-nanovaccine activated DCs, and induced a Th1 type of immune response. It was further observed that MUC4β-nanovaccine-immunized mice produced more IgG2b anti-MUC4β antibodies than IgG1 antibodies, suggesting that MUC4β-nanovaccine induces sufficient IFN-γ to promote antibody isotype switching consistent with a Th1-like immune response. Therefore, the recombinant human MUC4β-based polyanhydride nanovaccine has the potential to be an effective immunotherapeutic modality against PC and other MUC4-overexpressing malignancies.

## RESULTS

### Encapsulation of MUC4β into polyanhydride NPs provides sustained antigen release

**Figure 1:**
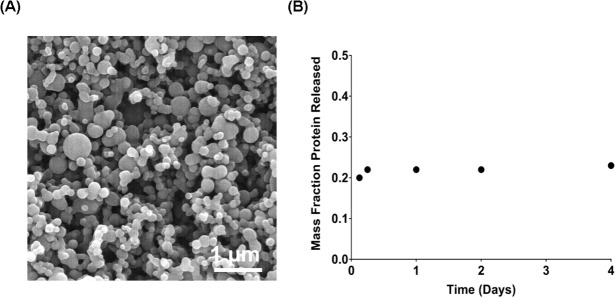
Encapsulation and release kinetics of MUC4β-nanovaccine Endotoxin-free recombinant MUC4β protein was isolated from *E. coli* Rosetta^TM^ 2 (DE3) strain bacteria and purified by affinity chromatography. 20:80 CPTEG:CPH NPs encapsulating 3% MUC4β were synthesized *via* a solid/oil/oil double emulsion flash nanoprecipitation process. **A.** Scanning electron microscope images of 20:80 CPTEG:CPH NP-encapsulated recombinant : MUC4β. **B.** Antigen release kinetics were characterized by incubating the NPs in PBS and measuring MUC4β released at regular intervals with a microBCA assay. 3% MUC4β-loaded 20:80 CPTEG:CPH NPs exhibited an initial burst (20%) release of protein followed by sustained release. The encapsulation efficiency of protein was determined to be ~32%.

The 20:80 CPTEG:CPH NPs loaded with 3% wt/wt MUC4β (endotoxin level < 0.01EU/mg) were synthesized *via* solid-oil-oil double emulsion. Scanning electron microscopy showed the NPs to be relatively spherical with a geometric mean diameter of 147 nm (with a geometric standard deviation of 1.3) (Figure 1A). The release kinetics of MUC4β from 20:80 NPs showed a burst of approximately 20% at early time points followed by slow and sustained release of protein (Figure 1B). The data showed that after four days, the 20:80 CPTEG:CPH particles released 23% of the encapsulated protein in a near-zero order release profile, which was consistent with previous work on protein release kinetics from CPTEG:CPH polyanhydride formulations [23, 24, 28–30]. Finally, the encapsulation efficiency of the MUC4β was determined to be 32 ± 1%.

### MUC4β-nanovaccine enhances the surface expression of MHC and co-stimulatory molecules on DCs

**Figure 2:**
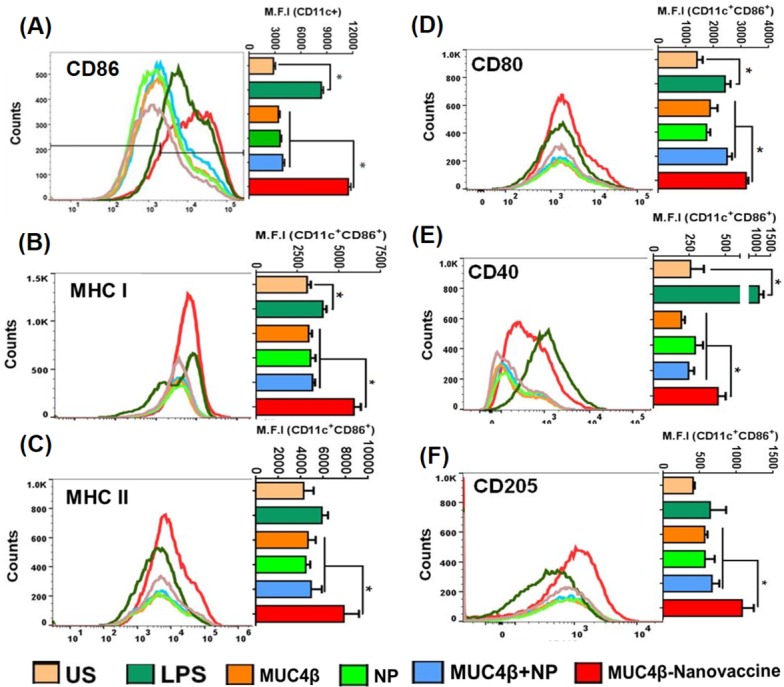
MUC4β-nanovaccine activates DCs and induces expression of MHC II and co-stimulatory molecules Flow cytometry analysis of *in vitro* antigen-pulsed DCs shows that MUC4β-nanovaccine activates DCs robustly as compared to other treatment groups. **A.** MUC4β-nanovaccine significantly upregulates CD86 expression in the CD11c^+^ population as analyzed by comparing the Mean Fluorescence Intensity (MFI) among all the treatment groups. LPS served as a positive control in the experiment, and unstimulated (US) DCs as negative control. **B-F.** Analysis of costimulatory and activation markers in CD11c^+^CD86^+^ cells. MUC4β-nanovaccine treatment upregulated the expression levels of MHC I **(B)**, MHC II **(C)**, CD80 **(D)**, CD40 **(E)**, and CD205 **(F)** after 48 h of stimulation using MUC4β protein (3 μg/mL), NP (100 μg/mL), or MUC4β+NP and MUC4β-nanovaccine (100 μg/mL). The fluorophore-conjugated antibodies were used at a 1:300 dilution except anti-CD40 antibody, which was used at a 1:200 dilution. The representative histograms for each surface marker have been presented with adjacent bar graphs showing the post-treatment MFI change. Each histogram plot represents a single experiment and the MFI plots summarize all experiments (*n* = 3). One-way ANOVA was used for data comparison for each group and pair wise comparison was performed using Student's paired t-test. The statistical significance was set at *p* < 0.001.

While the functional role of MUC4 in PC pathobiology has been studied extensively [16, 31–38], the utility of MUC4 as a candidate for vaccine development remains to be explored systematically. To examine the antigenicity of MUC4β and characterize the potential of MUC4β-nanovaccine in activating mature DCs, flow cytometry was used to measure the expression of cell surface markers such as MHC II and MHC I, co-stimulatory molecules CD80 and CD86, CD40, and C-type lectin CD205 (DEC-205, a DC maturation marker). After 9 days of IL-4 and GM-CSF treatment, more than 60% of the bone marrow-derived cells in culture differentiated into CD11c^+^ DCs. MUC4β-nanovaccine resulted in significantly higher activation of DCs as compared to other treatments as shown by the increased levels of costimulatory marker CD86 on the CD11c^+^ population (Figure 2A). There was no significant difference in the proportion of CD11c^+^CD86^+^ cells (activated DCs) across various treatments with the exception of cells treated with LPS as a positive control (Supplementary Figure 1). However, an increase in mean fluorescence intensity (MFI) indicated that the MUC4β-nanovaccine upregulated DC activation markers on the CD11c^+^CD86^+^ population as compared to the unstimulated control and other treatment groups. On the CD11c^+^CD86^+^ gated cells, there was a significant upregulation of MHC I, CD80, and CD40 following LPS treatment. As compared to the unstimulated control and other treatment groups (MUC4β alone, NP alone, and MUC4β+NP), treatment with MUC4β-nanovaccine significantly enhanced the expression of MHC I, MHC II, and CD40 (Figure 2B-2E). As compared to unstimulated control, MUC4β-nanovaccine resulted in a 1.9-fold increase in MHC I and a 1.83-fold increase in MHC II on CD11c^+^CD86^+^cells (Figure 2B-2C). Similarly, there was a significant increase in the expression of CD80 on CD11c^+^CD86^+^ cells following treatment with MUC4β-nanovaccine (2.25-fold) and MUC4β+NP (1.76-fold) as compared to untreated control, while treatment with MUC4β alone or blank NPs had no effect (Figure 2D). Further, as compared to the unstimulated control, MUC4β-nanovaccine treatment significantly enhanced the surface expression of CD40 (1.72-fold; *p* < 0.001) on CD11c^+^CD86^+^cells, while there was no change following treatment with MUC4β alone, blank NP or MUC4β+NP (Figure 2E). Lastly, the expression of CD205 was significantly upregulated on MUC4β-nanovaccine-stimulated DCs (2.6-fold; *p* < 0.001) when compared to the unstimulated control group (Figure 2F). These results suggest that the MUC4β-nanovaccine upregulates MHC I, MHC II, and costimulatory molecules and promotes antigen presentation by DCs i *in vitro*.

### MUC4β-nanovaccine induces pro-inflammatory DC cytokine secretion

**Figure 3:**
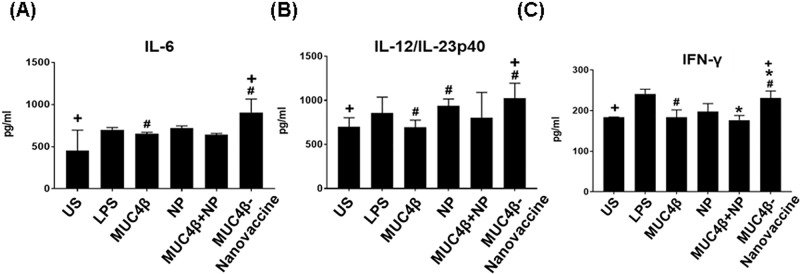
MUC4β-nanovaccine induces Th1 DC cytokine secretion Cytokine analysis demonstrated that MUC4β-nanovaccine-activated DCs secrete Th1 cytokines. Free MUC4β mixed with empty NPs (MUC4β+NP)-pulsed DCs and MUC4β -pulsed DCs produced Th1 cytokines at levels compared to the unstimulated (US) DCs. Only encapsulation of MUC4β could modulate and reprogram DCs to secrete higher levels of Th1 cytokines IL-6 **(A)**, IL-12/IL-23p40 **(B)**, and IFN-γ **(C)**. For each treatment group, the sample size was *n* = 4. Statistical significance was set at *p* < 0.05. ANOVA analysis of the data was *p* < 0.05. Statistical comparison between MUC4β, MUC4β+NP and US with MUC4β-nanovaccine is denoted by #, ^*^ & + respectively.

DCs direct immune responses not only by interacting with lymphocytes and presenting antigen [39, 40], but also by secreting an array of cytokines that modulate these responses. The supernatant of DCs cultured with MUC4β-nanovaccine contained significantly higher levels of IL-12p40, IL-6, and IFN-γ as compared to supernatants from untreated DCs or DCs stimulated with MUC4β alone or MUC4β+NP (Figure 3). The amounts of IL-6 and IL-12/ IL-23p40 in culture supernatants treated with MUC4β-nanovaccine were 2-fold and 1.5-fold higher, respectively, as compared to the unstimulated control (Figure 3A & 3B). The level of IFN-γ after MUC4β-nanovaccine treatment was found to be 1.25-fold higher than unstimulated control groups (Figure 3C). DCs treated with MUC4β alone or MUC4β+NP expressed low levels of cytokines, which were not significantly different than the levels expressed by unstimulated DCs. Similar to enhanced surface expression of MHC II and co-stimulatory molecules, DCs secreted higher levels of cytokines when treated with encapsulated MUC4β in 20:80 CPTEG:CPH NPs.

### Immunization with MUC4β-nanovaccine elicits robust anti-MUC4 humoral responses

**Figure 4:**
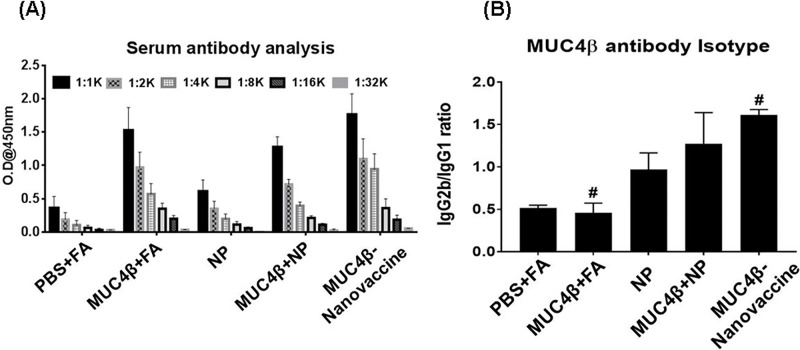
MUC4β-nanovaccine generates anti-MUC4β Th1 humoral response in mice **A.** Titers of anti-MUC4β antibodies were determined by ELISA in the sera of mice immunized with various formulations, and suggest that MUC4β emulsified in Freund's adjuvant (MUC4β +FA), or mixed with empty nanoparticles (MUC4β+NP), or encapsulated in the nanoparticles (MUC4β-nanovaccine)-elicited anti-MUC4 antibodies. **B.** Isotyping of anti-MUC4β antibodies (at 1:1000 dilution) demonstrates that isotype switching was predominantly to Th1 IgG2b in MUC4β-nanovaccine-immunized mice, whereas in MUC4β-immunized mice it was predominantly the Th2 IgG1 isotype. For each treatment group sample size was *n* = 3. Statistical significance was set at *p* < 0.05. ANOVA analysis of the data was *p* < 0.05. Statistical comparison between MUC4β+FA and MUC4β-nanovaccine is denoted by #.

Polyanhydride nanovaccines have been shown to induce the formation of germinal center B-cells that result in sustained serum antibody responses after a single dose [41]. The presence of high levels of antigen-specific IgG2b over IgG1 indicates isotype-switching mediated by IFN-γ, whereas a low IgG2b:IgG1 ratio is indicative of Th2-like T-cell-response [42]. To investigate if immunization with MUC4β-nanovaccine induced robust humoral immune responses, animals were immunized subcutaneously twice (days 0 and 14) following the prime-boost regimen with MUC4β-nanovaccine or various control formulations (MUC4β emulsified in Freuend's adjuvant or MUC4β free protein mixed with empty NPs). Five days after the booster, the anti-MUC4β titer was 16,000 in the sera of mice that were immunized with the three formulations containing MUC4β. However, the reactivity was comparable and higher in mice administered with MUC4β emulsified in Freund's adjuvant (FA) and MUC4β-nanovaccine as compared to the mice that received a mixture of free protein and blank NPs (Figure 4A). Importantly, mice immunized with the MUC4β-nanovaccine demonstrated a higher IgG2b:IgG1 ratio than mice immunized with MUC4β+FA (Figure 4B). These results indicate that MUC4β protein encapsulated in 20:80 CPTEG:CPH NPs predominantly elicits a Th1-type immune response.

### Immunization with MUC4β-nanovaccine enhances the circulating levels of inflammatory cytokines

**Figure 5:**
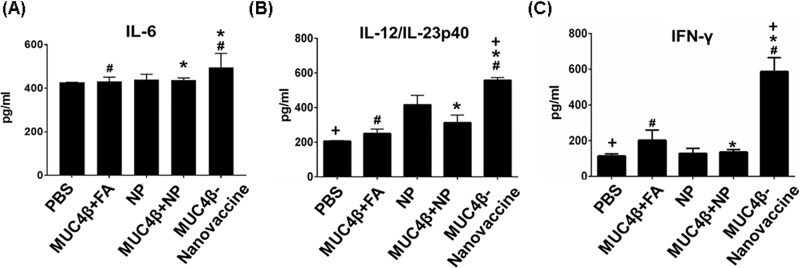
Presence of pro-inflammatory cytokines in the sera of mice immunized with MUC4β-nanovaccine Levels of IL6 (**A**), IL-12 **(B)** and IFN-γ (**C**) in the sera of mice with immunied with various formulations. The sera of MUC4β-nanovaccine-immunized mice had significantly higher levels of IL6, IL-12/IL23p40, and IFN-γ as compared to mice immunized with MUC4β+FA, and MUC4β+NP. For each treatment group, the sample size was *n* = 3. Statistical significance was set at *p* < 0.05. ANOVA analysis of the data was *p* < 0.05. Statistical comparison between MUC4β+FA, and MUC4β+NP with MUC4β-nanovaccine is denoted by # & ^*^ respectively.

Next, the presence of pro-inflammatory cytokines (IL-6, IL-12, and IFN-γ) in the sera of immunized mice was investigated. Sera from MUC4β-nanovaccine-immunized mice had significantly higher levels of IL-6 compared to MUC4β+FA- and MUC4β+NP-immunized mice (Figure 5A). The sera from MUC4β-nanovaccine-immunized mice had significantly higher amounts of IL-12/IL-23p40 (~2.7-fold increase) and inflammatory cytokine IFN-γ (~5.2-fold increase) when compared to PBS-treated control mice (Figure 5B, 5C). Similarly, as compared to sera from the mice immunized with MUC4β+FA, we observed a significant increase of IL-12/ IL-23p40 (~2.2-folds) and IFN-γ (~4.5-folds) cytokines in the sera of MUC4β-nanovaccine-immunized mice (Figure 5B, 5C). Significant upregulation of IFN-γ, as compared to control group as well as other treatment groups (Figure 5C), suggests that MUC4β-nanovaccine promotes Th1 mediated isotype switching of antibodies in these mice.

## DISCUSSION

To date vaccine development involving mucins has been based on selected peptides that have a limited repertoire of immunogenic epitopes and have predominantly focused on the tandem repeat domains which are typically O-glycosylated. Recombinant proteins could address these limitations by presenting the entire spectrum of possible epitopes present in the original antigens in an unbiased manner [3]. In this study, the β-subunit of MUC4 expressed and purified from a bacterial expression system was used to investigate its potential as an effective immunogen. The data presented show that free MUC4β alone induces relatively low expression of MHC I and MHC II complexes, co-stimulatory molecule CD80 (Figure 2B-2D), and inflammatory cytokines in DCs (Figure 3A-3C) *in vitro*. Immunization of mice with MUC4β in combination with Freund's adjuvant likely induces a Th-2 like response based on the induction of significantly higher levels of IgG1 antibodies (Figure 4). Th2 immune responses have been well established to promote tumor pathogenesis and aggressiveness, whereas shifting the immune response to Th1 phenotype provides anti-tumor protection [43–45]. In this study, MUC4β was encapsulated into 20:80 CPTEG:CPH nanoparticles (MUC4β-nanovaccine) in order to investigate whether the MUC4β-nanovaccine could enhanced activation of DCs and modulated Th1-mediated humoral responses.

The MUC4β-nanovaccine enhanced surface expression of MHC I and MHC II on CD86^+^CD11c^+^ DCs (Figure 2B-2C), which are implicated in presentation of antigen, and increased the surface expression of CD80 (Figure 2D), a co-stimulatory molecule required for activation T-cells. Antigen that is taken up *via* CD205 enters the MHC I and MHC II antigen presentation pathways, and thus an increase in CD205 expression enhances antigen presentation by DCs to both CD8+ cytotoxic T-cells and CD4^+^ helper T-cells and could potentially lead to long-lived immunity by specifically targeting the cancer cells. [46, 47]. MUC4β-nanovaccine significantly enhanced surface expression of CD205 on DCs (Figure 2F) that corresponded with increased expression of MHC complexes on these DCs. Thus, upregulation of these markers by MUC4β-nanovaccine, in comparison to free MUC4β-pulsed DCs, suggests that the encapsulated formulation was better in potentiating antigen presentation by DCs. Further, it was observed that only stimulation with MUC4β-nanovaccine enhanced DC secretion of the pro-inflammatory cytokines IL-6, IL-12/IL-23p40, and IFN-γ *in vitro* (Figure 3) as compared to free MUC4β protein or MUC4β mixed with empty NPs (MUC4β+NP), which corroborated the observed alterations in the expression of DC activation markers. These results indicate that the encapsulation of MUC4β protein in CPTEG:CPH NPs is crucial in stimulating a cytokine profile that would enhance a Th1-like immune response.

Previously, it has been shown that a single immunization of polyanhydride nanovaccines can induce high antibody titers and provide protective immunity against *Yersinia pestis* in mice [29, 48]. Additionally, it is important to consider the quality of the antibody response generated by the nanovaccine, which may be characterized by the specificity, avidity and isotype profile of the antibody response [49]. It is therefore noteworthy that the MUC4β-nanovaccine-immunized mice had the highest IgG2b:IgG1 ratio, which indicates induction of isotype class switching, whereas MUC4β+FA or MUC4β in combination with blank NPs preferentially induced IgG1 anti-MUC4β antibodies (Figure 4). This supports our *in vitro* observation that encapsulation of MUC4β into polyanhydride NPs likely plays a crucial role in activating DCs to produce cytokines (IL-6, IL-12/ IL-23p40 and IFN-γ) (Figure 3) that would polarize the immune response towards a Th1 phenotype. This was further validated with the detection of higher levels of IFN-γ in the sera of MUC4β-nanovaccine-immunized mice (Figure 5C). The data herein shows encapsulating MUC4β into 20:80 CPTEG: CPH NPs is an effective strategy to activate DCs and modulate the response towards an anti-MUC4 Th1 phenotype and potentially generate antigen-specific cytotoxic CD8^+^ T-cells. IgG2b antibodies may possibly provide additional immunity against MUC4-expressing tumors by inducing antibody-dependent-cellular cytotoxicity [50, 51]. The current studies provides a basis for investigating the use of the MUC4β-nanovaccine as an immunotherapeutic strategy in cancer models that overexpress MUC4 as a tumor-associated antigen.

## MATERIALS AND METHODS

### MUC4β purification

The cDNA sequence encoding the 733 amino acids (2199 base pairs) of human MUC4β fragment was cloned into the bacterial expression vector pET-28a (Novagen, USA), and transformed into the *E. coli* Rosetta^TM^ 2 (DE3) strain. Bacterial culture was grown under standard conditions and MUC4β was purified by AKTA Ni-NTA affinity chromatography. Eluted fractions (in 6M urea) containing the recombinant MUC4β protein were assessed with SDS-PAGE gels and further confirmed with immunoblotting. All the verified fractions were pooled and concentrated using Amicon ultracentrifugal filters (50 kDa molecular weight cut-off). A step-wise dialysis was performed against a decreasing concentration of urea in 1Χ PBS to allow the protein refolding. Purified fractions were passed through an endotoxin removal spin column (Pierce). Finally, the concentration of our target protein and the level of endotoxins in the samples were measured using the BCA Protein Assay Kit (Pierce Thermo Fisher) and the Pierce LAL Chromogenic Endotoxin assay kit, respectively.

### Encapsulation of MUC4β in polyanhydride nanoparticles

The 20:80 CPTEG:CPH polymer was synthesized *via* melt polycondensation [24]. Next the nanoparticles encapsulating MUC4β were synthesized using a solid-oil-oil double emulsion technique as described previously [48]. Briefly, purified MUC4β was dialyzed to nanopure water and lyophilized. Next, 20:80 CPTEG:CPH polymer containing 3% wt. of MUC4β was dissolved at 20 mg/ mL in methylene chloride. The solution was sonicated for 30 s to ensure even distribution of the protein. The nanoparticles were precipitated into chilled pentane (−10°C; 1:250 methylene chloride: pentane) and collected *via* vacuum filtration. NP morphology was verified by scanning electron microscopy (FEI Quanta 250, FEI, Hillsboro, OR) and NP size subsequently analyzed with ImageJ (ImageJ 1.48v, NIH).

The release kinetics of MUC4β from 20:80 CPTEG:CPH nanoparticles were monitored as previously described [23]. Briefly, NPs were incubated in PBS at 37°C. Periodically, the samples were centrifuged, supernatant collected, and particles were resuspended in fresh buffer. The amount of protein in the collected supernatant was quantified using a microBCA assay. Further, the encapsulation efficiency was determined by comparing the total amount of protein released to the amount theoretically encapsulated. Briefly, at the end of approximately one month, the buffer was exchanged with 40 mM sodium hydroxide to quickly degrade the NPs and release any remaining protein, which was estimated using microBCA assay.

### Primary dendritic cells (DCs) isolation

C57BL/6 mice were kept under specific pathogen-free conditions at the UNMC Animal Facility in accordance with UNMC Institutional Animal Care and Use Committee (IACUC) standards. Femurs and tibiae of 6-8 week old C57BL/6 mice were removed and the marrow was flushed in RPMI medium using a 1 mL syringe attached to a 25 G needle. Cells were centrifuged at 2000 rpm for 2.5 min and the cell pellet was resuspended in 10 mL of 1x RBC lysis buffer in the dark for 5 min. Ten mL RPMI medium was added to stop the lysis. Cells were centrifuged at 2000 rpm and washed 3 times in 10 mL of RPMI medium. After the last wash, cells were resuspended homogeneously in a 1 bone: 1 mL of media ratio. Resuspended cells were passed through a cell strainer to remove clumps. Cell suspensions were poured into 10 mL RPMI medium containing 10% FBS, penicillin (100 U/mL, Sigma) and streptomycin (100 mg/mL, Sigma) in a 10 cm tissue culture plate that was then kept in an incubator for 3 h. After incubation, all nonadharent cells were collected and the plate was washed with medium twice to collect any remaining nonadharent cells into a 50 mL tube. The cells were pelleted down by centrifugation and the medium aspirated. The cells were resuspended in 10 mL complete RPMI medium containing 50 μM β-merceptoethanol (β-ME), 1 mM sodium pyruvate, and 10 mM HEPES and transferred into T75 flasks for our *in vitro* studies.

### DC maturation and pulsing

To generate bone marrow derived DC population, we added 50 ng/mL of recombinant mouse (rm) GM-CSF and 25 ng/mL rm-IL-4 reconstituted and diluted in serum-free RPMI medium to freshly isolated DC cultures on Day 0. On Day 3, 5, and 7, the non-adherent cells were collected in 50 mL centrifuge tubes, pelleted them and re-suspended in a total volume of 10 mL of fresh RPMI medium containing 10% FBS, penicillin and streptomycin antibiotics, 10 mM HEPES, 1 mM sodium pyruvate, 50 μM β- ME, 50 ng/mL of GM-CSF and 25 ng/mL IL-4 in a fresh 6-well plate. At Day 9, immature DCs were counted and seeded in a 24 well plate for activation studies. Polyanhydride NPs were suspended in complete culture medium, sonicated briefly (30 s on ice), and added to the DC cultures at Day 9 at a concentration of 100 μg/mL. DCs were pulsed in the following groups: 3 μg/mL free MUC4β protein, free MUC4β protein (3 μg/mL) mixed with blank NPs (100 μg/mL) (MUC4β+NP) and MUC4β-nanovaccine (100 μg/mL). Unstimulated DCs (US) and DCs stimulated with LPS (200 ng/mL) were used as negative and positive controls, respectively. Cultures were incubated for 48 h (37°C, 5% CO_2_). Activated DCs were harvested from the 24-well plate and centrifuged at 1000 x g for 5 min to collect the culture medium and the pellet was processed further for flow cytometry studies. Supernatant was collected for ELISA studies from each treatment group.

### Flow cytometry of activated DCs

DCs were resuspended and washed 3 times in FACS buffer containing 1xPBS (pH 7.2) and 1% fetal bovine serum (FBS) to remove any residual culture medium. After that, the 1X10^5^ DCs were resuspended in a 100 μL volume of conjugated antibody cocktail for detection of DC surface markers consisting of antibodies recognizing CD11c, MHC I, MHC II, CD80, CD86, and CD205 and prepared at a 1:300 dilution except anti-CD40 antibody, which was used at 1:200 dilution in FACS Buffer. Corresponding isotype controls were also prepared at similar dilutions in FACS buffer. Following incubation with antibodies for 60 min on ice (4°C) in the dark, the cells were washed thrice with FACs buffer, and fixed with 4% paraformaldehyde for 15 min at RT. The cells were washed again and analyzed using a BD LSR-II Green Flow Cytometer (BD Biosciences). FlowJo® and BD FACSDIVA software were used to analyze the data.

### Cytokine analysis by ELISA

Supernatants were preserved at −80°C. IL-6, IFN-γ and IL-12/IL-23 p40 cytokines were measured by enzyme-linked immunosorbent assay (ELISA) kits from BioLegend and the manufacturer's protocol was followed for the assay. 96-well ELISA strips were coated with capture antibodies (1:200 dilution) in carbonate-bicarbonate buffer (0.5M, pH 9.6) and incubated overnight at 4°C. The next day coated strips were washed 4 times with 1xPBST (1x PBS and 0.05% Tween 20) and blocked with 3% bovine serum albumin (BSA) in PBS for 2 hours at 37°C. Strips were then washed 4 times and 100 μL of supernatants were added to coated strips and incubated for 2 hours at 37°C. Plates were washed with PBST for 4 times, followed by incubation with detection antibody (1:200) for 1 hour at 37°C. Excess secondary antibody was washed away with 4 PBST washes. Avidin (1:1000) was added to ELISA strips and incubated for 30 min at RT in dark. Excess avidin was removed with 5 PBST washes, followed by addition of TMB (3,3′,5,5′-Tetramethylbenzidine) substrate in the dark and the strips were incubated at RT for color to develop. Absorbance was measured after the reaction was stopped (~15 min) with 1N H_2_SO_4_ at 450 nm using a SpectraMax® Plus384 microplate reader (Molecular Devices LLC, Sunnyvale, California). For serum cytokines analysis, serum samples were collected post-euthanasia and stored at −80°C. Serum samples were added at 1:10 dilution to capture-antibody coated plates for cytokine analysis.

### Mice immunization

Eight-week old C57BL/6 mice were immunized subcutaneously with various formulations of recombinant MUC4β protein emulsified with Freund's incomplete adjuvant (MUC4β+FA: 20 μg/mouse/dose), encapsulated MUC4β protein (MUC4β-nanovaccine: 20 μg protein+300 μg NP/mouse/dose), protein plus empty NPs (MUC4β+NP: 20 μg MUC4β+300 μg empty NP / mouse/dose), and PBS+ Freund's adjuvant control. A booster immunization was given 2 weeks after the primary immunization and blood was collected from the submandibular vein after 5 days and processed for serum preparation.

### Anti-MUC4β antibodies detection in immunized mice sera by ELISA

To detect anti-MUC4β antibodies in the sera of immunized mice, we used a modified ELISA protocol. A 96-well plate was coated with 5 μg/mL of recombinant MUC4β protein in carbonate-bicarbonate coating buffer (0.5 M, pH 9.6) and incubated overnight at 4°C. The plate was washed 2 times with 1xPBST and blocked with 3% BSA in PBS for 3 hours at 37°C. Further, the plate was washed 4 times and then serial dilutions of the serum samples were incubated in a MUC4β-coated plate for 2 hours at 37°C. Plates were washed with PBST 4 times, followed by incubation with horseradish-peroxidase (HRP) conjugated goat anti-mouse IgG (total H+L) (Thermo Fisher), IgG1 (Abcam) or IgG2b (Abcam) for 1 hour at 37°C. Excess secondary antibody was washed away with 5 PBST washes followed by the addition of TMB substrate in the dark. Absorbance was measured after the reaction was stopped (at ~15 min) with 1N H_2_SO_4_ at 450 nm using a SpectraMax® Plus384 microplate reader (Molecular Devices LLC, Sunnyvale, California).

### Antibodies

Alexa Fluor® 700 anti-mouse CD11c (clone N418), anti-Mouse MHC Class I (H-2Kb) eFluor® 450 (Clone: AF6-88.5.5.3), FITC conjugated anti-mouse/rat MHC Class II (I-A/I-E; clone M5/114.15.2), APC anti-mouse CD40 (clone 1C10), PE conjugated anti-mouse CD80 (clone 16-10A1) antibodies with their respective isotype controls comprising of Alexa Fluor® 700 conjugated Armenian hamster IgG (clone eBio299Arm), FITC rat IgG2b κ (clone eB149/10H5), Rat IgG2a k Isotype Control APC (clone eBR2a), PE-conjugated rat IgG2a (clone eBR2a), Rat IgG2a k Isotype Control PE-Cyanine7 (clone eBR2a), and mouse IgG2a k Isotype Control eFluor® 450 (clone eBM2a) were purchased from eBioscience^™^ (San Diego, CA). PE/Cy7^™^ rat anti-mouse CD86 (Clone GL1) was purchased from BD Pharmingen^™^. PE/Cy 5.5 anti-mouse CD205 (MMR, clone NLDC-145) and PerCP/ Cy5.5 Rat IgG2a, κ Isotype control were procured from BioLegend®.

### Statistical analysis

Differences among group means were tested by one-way analysis of variance (ANOVA) F-test using GraphPad Prism v. 7.0 (GraphPad, La Jolla, CA). If the F-test was significant, Tukey's t-tests were performed for pairwise comparisons of group means. Significance was defined at *p* < 0.05 or *p* < 0.001 as indicated in the respective figure legends.

## SUPPLEMENTARY FIGURE


