# Supplementary material for: Amphiphilic polyanhydride-based recombinant MUC4β-nanovaccine activates dendritic cells
Source: Genes Cancer. 2019 May;10(3-4):52–62. doi: 10.18632/genesandcancer.189 (PMC6584211; doi:10.18632/genesandcancer.189)
Supplement: Supplementary file 1 [file ganc-10-052-s001.pdf]

Amphiphilic polyanhydride-based recombinant MUC4β-nanovaccine activates dendritic cells – Banerjee et al

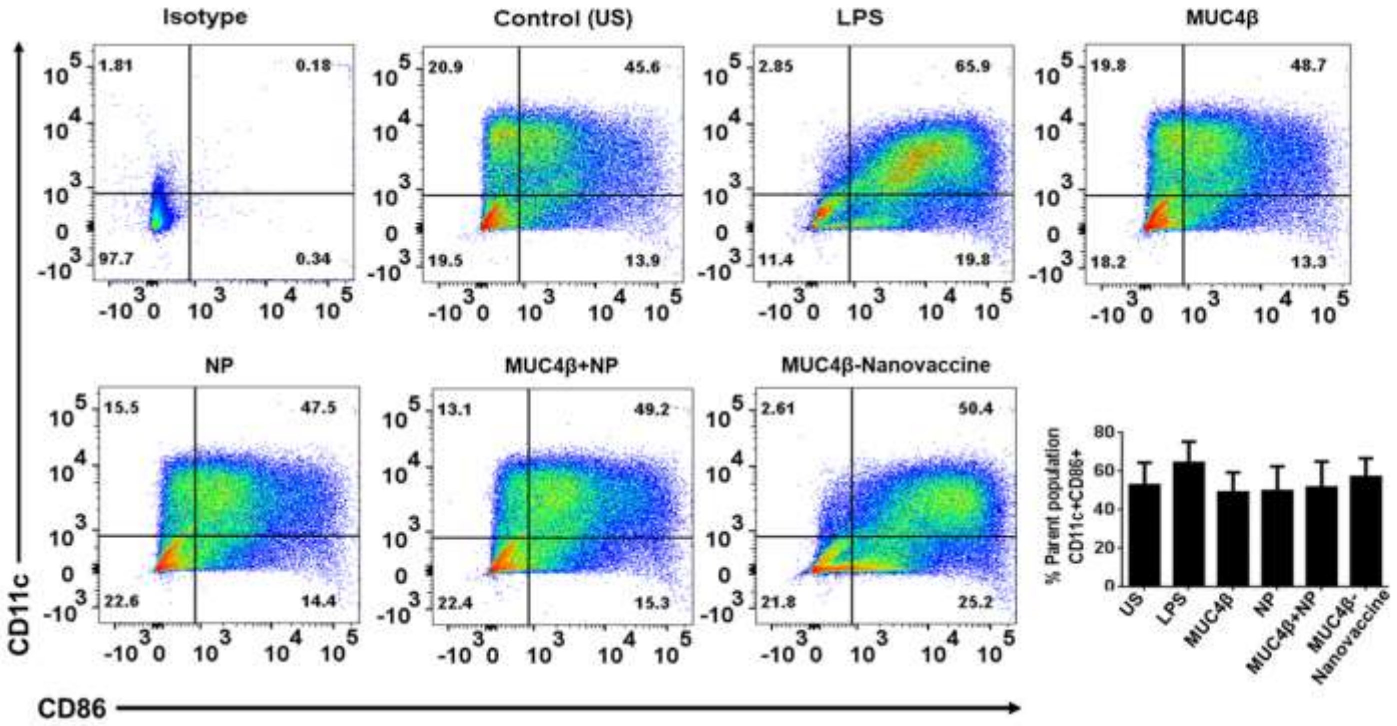

Supplementary Figure 1: Flow cytometry analysis of antigen-pulsed murine DCs for CD11c^{+}CD86^{+} population.

Antigen-pulsed DCs after 48 h of treatment with MUC4β, NP, MUC4β+NP, and MUC4β-nanovaccine were analyzed for DC surface marker CD11c (y-axis) in combination with costimulatory marker CD86 (x-axis) using flow cytometry. Representative dot plot has been shown in the figure. CD11c^{+}CD86^{+} population (Q2) is used in further data analysis for different surface markers in various treatment groups. The proportion of CD11c^{+}CD86^{+} cells in the total population following various treatments is presented in the bar graph in the lower panel.
